# Comparative BRAF V600E immunohistochemical expression in differentiated thyroid tumors with papillary features

**DOI:** 10.25122/jml-2021-0415

**Published:** 2022-04

**Authors:** Maha Hatem Abdul Wahid, Rihab Hameed Almudhafar

**Affiliations:** 1. Department of Basic Sciences, Faculty of Dentistry, University of Kufa, Kufa, Iraq; 2. Middle Euphrates Unit for Cancer Research, Faculty of Medicine, University of Kufa, Kufa, Iraq

**Keywords:** DTC, BRAF, immunohistochemistry, DTC – differentiated thyroid carcinoma, E-FVPTC – Encapsulated follicular variant papillary thyroid cancer, FVPTC – Follicular variant of papillary thyroid carcinoma, I-FVPTC – Infiltrative follicular variant papillary thyroid cancer, IHC – Immunohistochemistry, MAPK – Mitogen-activated protein kinase, NIFTP – Non-invasive follicular thyroid neoplasm with papillary-like nuclear features, PTC – Papillary thyroid carcinoma, PTMC – Papillary thyroid microcarcinoma

## Abstract

Differentiated thyroid tumors (DTC) are the most common indolent tumors associated with a good prognosis compared with other tumors. Its incidence during the last few decades has increased. DTC includes papillary carcinoma and follicular carcinoma. The BRAF is the most prevalent genetic mutation in thyroid carcinoma, occurring in more than 50% of papillary thyroid cancers (PTCs). The study aimed to evaluate BRAF expression in differentiated thyroid tumors with papillary-like nuclear features. Formalin-fixed paraffin-embedded blocks (FFPE) were collected from archival samples of patients in private histopathology labs in Al-Najaf city from 55 cases, which included 27 papillary thyroid carcinoma (PTC) cases, 10 cases of NIFTP, 13 FVPTC cases, 2 papillary microcarcinoma cases, and 3 NIFTP coexist with papillary microcarcinoma cases. All samples were stained using the immunohistochemistry method in the Middle Euphrates unit for cancer research at the University of Kufa/Faculty of Medicine. 15/55 (27.3%) of cases increased BRAF expression. The BRAF expression was statistically significant with tumor type (p=0.008). The higher expression was associated with 13 (48.15%) of PTC cases. However, the BRAF expression did not correlate with gender (p=0.2), tumor size (p=0.07), and tumor focality (p=0.09). BRAF V600E has prognostic value as it correlates with tumor progression.

## Introduction

Papillary thyroid cancer (PTC) is considered a common histological type of thyroid malignancy. PTC carries a good prognosis with up to 95% survival rate after ten years [1]. In Iraq, according to cancer registration from the Ministry of Health in 2012, thyroid cancer was the 7^th^ common malignant tumor among the ten most common malignancies in females, which accounts for 3.76% [2]. There are two most common variants of PTC, including the follicular variants and classic variants [3]. PTC was identified based on the characteristic nuclear features such as chromatin margination, nuclear enlargement, nuclear overlapping, intra-nuclear pseudo-inclusions, and nuclear grooves [4]. In 2006, the follicular variant of papillary thyroid carcinoma (FVPTC) was classified into two types [5]: infiltrative follicular variant papillary thyroid cancer (I-FVPTC) and encapsulated follicular variant papillary thyroid carcinoma (E-FVPTC) [6]. Infiltrative FVPTC tumors typically behave similarly to classical papillary thyroid carcinoma [7]. The encapsulated FVPTC (EFVPTC) behaves more similarly to a follicular adenoma [8–9]. Papillary thyroid microcarcinoma (PTMC) is characterized as papillary thyroid carcinoma with a 10 mm diameter or less [10]. Over the last few years, it has been increasing in incidence and accounts for nearly half of the rise in papillary thyroid cancer [11, 12]. PTMC is considered an indolent illness in general, but it does carry the risk of distant metastasis and local recurrence [13]. Non-invasive follicular thyroid neoplasm with papillary-like nuclear features (NIFTP) represented the indolent behavior of thyroid neoplasm [14], with a low risk of relapse [15]. NIFTP is a non-invasive thyroid neoplasm of follicular cell derivation characterized by nuclear features of PTC and follicular growth pattern and has an extremely low malignant potential [8]. NIFTP is neither definitely benign nor definitely malignant tumor [16]. BRAF mutations are common in both benign and malignant human tumors. According to the Catalogue of Somatic Mutations in Cancer, it is estimated that 5–7% of all human neoplasms have BRAF alterations [17]. The BRAF is the most prevalent mutation in thyroid cancer, which occurs in more than 50% of papillary thyroid cancers (PTCs) and approximately 45% of anaplastic thyroid cancer [18]. Asian populations have a higher BRAF mutation rate than Western countries [19].

## Material and Methods

The study was conducted in Iraq at Al-Najaf city, in the Middle Euphrates Unit for Cancer Research at the University of Kufa, Faculty of Medicine from September 2020 to August 2021. All included tumors were reexamined microscopically by two pathologists to confirm the diagnosis. The hematoxylin and eosin-stained sections from each case were revised concerning the pathological type to prove the diagnosis. The cases were classified according to the World Health Organization (WHO) classification of thyroid tumors.

### Primary antibody

Rabbit monoclonal BRAF V600E (BIO-SB, USA) is ready to use (RTU) for an *in vitro* diagnostic medical device (IVD).

### BRAF En Vision complex Immunohistochemistry

Positive and negative control slides were added with each run. We utilized the EnVision complex IHC technique using 4 microns of tissue sections from the formalin-fixed paraffin-embedded blocks. We continued with the deparaffinization and antigen retrieval step (AR) by using the water bath method at 90–95°C for 20 minutes, followed by adding a peroxidase-blocking reagent. Next, BIO-SB BRAF V600E primary antibody for 24 hours in a humid container, then HRP polymer was added for 30 minutes, followed by substrate chromogen and hematoxylin for 2 minutes. Each step was separated by rinsing with TBS.

### Statistical analysis

Data were analyzed using SPSS program version 26. The Chi-square test and Fisher exact test were used to assess the relationship between categorical variables such as focality. At the same time, continuous variables were presented as mean and standard deviation (SD). Kruskal-Wallis test was used for comparing the tumor size as it was not normally distributed.

## Results

As shown in Table 1, 15/55 (27.3%) of cases had increased BRAF expression. The BRAF expression was significantly associated with tumor type (p=0.008), and the higher expression was associated with 13 (48.15%) PTC cases.

**Table 1.** The correlation between BRAF and tumor types.

	**BRAF**		**Total**	**P**
	**Positive**	**Negative**		
**PTC**	(13) 48.15%	(14) 51.85%	(27) 100%	0.008
**Papillary micro carcinoma**	(1) 50%	(1) 50%	(2) 100%	
**FVPTC**	(1) 7.69%	(12) 92.31%	(13) 100%	
**NIFTP**	(0) 0%	(10) 100%	(10) 100%	
**NIFTP + Papillary micro carcinoma**	(0) 0%	(3) 100%	(3) 100%	
**Total**	(15) 27.3%	(40) 72.7%	(55) 100%	

As shown in Table 2, gender is an important clinicopathological parameter but did not reach statistical significance (p=0.2). The mutated BRAF V600E is more common in females 48/55(87.3%) than males. Also, the study results did not show a significant relationship between BRAF V600E and age (p=0.7). Larger tumor sizes associated with cases of NIFTP coexist with papillary microcarcinoma. Tumor size did not reach the level of statistical significance (p=0.07). In most cases, tumor focality is demonstrated as solitary in a large percentage of included cases. In malignant cases, PTC was documented as a solitary tumor in 17/27 (63%) of the cases. Tumor focality was not statistically significant (p=0.09).

**Table 2.** The correlation of tumor type with clinicopathological parameters.

	**BRAF V600E**	**Gender**		**P**	**Age group**		**P**	**Tumor size**	**P**	**Focality**		**P**
		**Male**	**Female**		**<35**	**≥35**				**Solitary**	**Multifocal**	
**PTC**	+BRAF	0 0%	13 48.15%	0.99	6 22.22%	7 25.93%	0.7	3.1±2.2	0.2	5 18.52%	8 29.63%	0.02
	-BRAF	1 3.7%	13 48.15%		8 29.63%	6 22.22%		2.2±1.4		12 44.44%	2 7.41%	
**Papillary micro carcinoma**	+BRAF	0 0%	1 50%	0.99	0 0%	1 50%	NA	0.9	NA	1 50%	0 0%	0.99
	-BRAF	1 50%	0 0%		0 0%	1 50%		1		0 0%	1 50%	
**FVPTC**	+BRAF	0 0%	1 7.7%	0.99	0 0%	1 7.7%	0.99	3	0.9	0 0%	1 7.7%	0.5
	-BRAF	2 15.38%	10 76.92%		6 46.15%	6 46.15%		3.1±1.9		7 53.84%	5 38.46%	
**NIFTP**	+BRAF	0 0%	0 0%	NA	0 0%	0 0%			NA	0 0%	0 0%	NA
	-BRAF	2 20%	8 80%		5 50%	5 50%		3.7±2.7		10 100%	0 0%	
**NIFTP + Papillary micro carcinoma**	+BRAF	0 0%	0 0%	NA	0 0%	0 0%	NA		NA	0 0%	0 0%	NA
	-BRAF	1 33.33%	2 66.67%		2 66.67%	1 33.55%		4.03±0.9		3 100%	0 0%	
**Total**		7 12.7%	48 87.3%		27 49.1%	28 50.9%		3±2.9		38 69.1%	17 30.9%	

**Figure 1.**
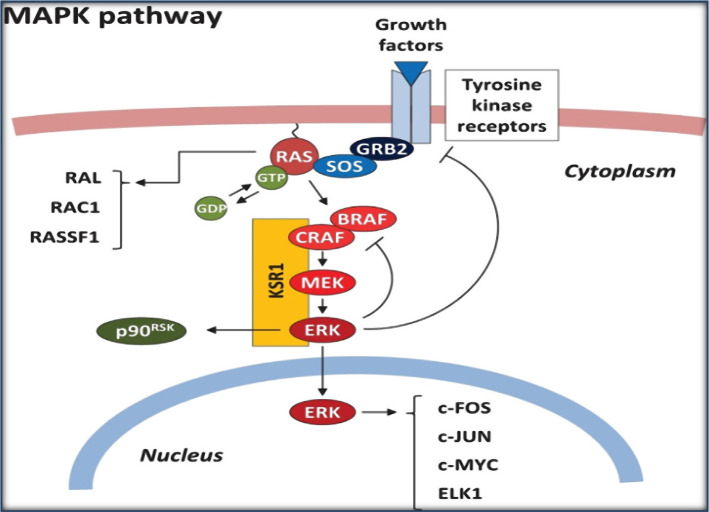
An overview of the Mitogen-activated signaling pathway kinase (MAPK) [20].

As illustrated in Figure 1, persistent activation of BRAF leads to activation of the mitogen-activated protein kinase (MAPK) pathway, which finally enhances tumor progression.

**Figure 2.**
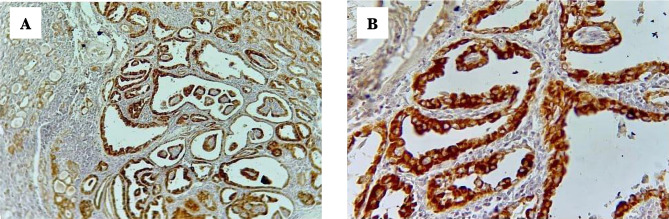
PTC, diffuse strong positive for BRAF V600E IHC (A) 10×10, (B) 10×40.

As shown in Figure 2, papillary thyroid carcinoma cases showed strong positive cytoplasmic stain of BRAF V600E immunohistochemistry, score index 12, and quick H score 300 with magnification power 10, 40, in (A) and (B), respectively.

**Figure 3.**
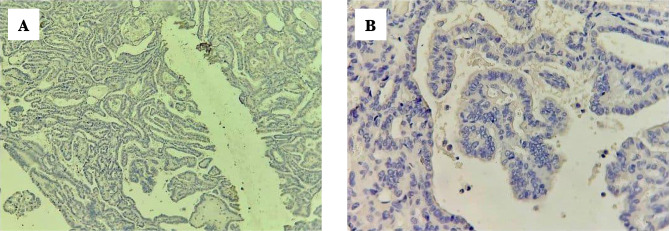
PTC, negative for BRAF V600E IHC. (A) 10×10, (B) 10×40.

In Figure 3 papillary thyroid carcinoma showed a negative cytoplasmic stain of BRAF V600E immunohistochemistry, score index 0, and quick H score 0 with magnification power 10, 40, in (A) and (B), respectively.

**Figure 4.**
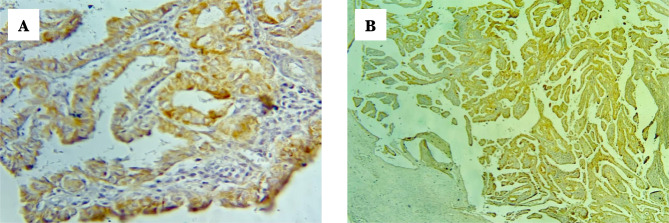
Papillary microcarcinoma, positive for BRAF V600E IHC (A) 10×4, (B) 10×40.

The papillary micro carcinoma demonstrated with positive BRAF V600E immunohistochemistry, score index 3, and quick H score 70 as demonstrated in Figure 4 in magnification power 10×4, 10×40, in (A) and (B), respectively.

**Figure 5.**
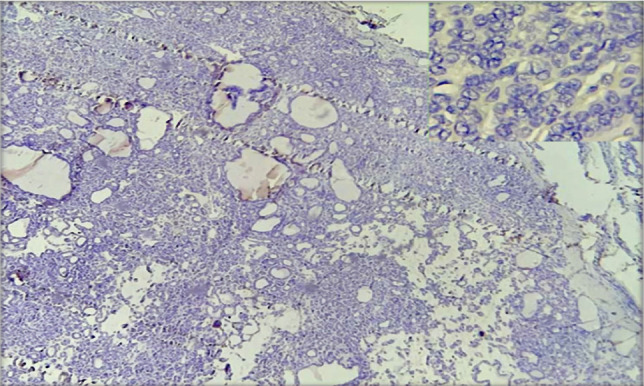
NIFTP, negative for BRAF V600E IHC (10×4).

The cytoplasmic stain of BRAF V600E immunohistochemistry showed negative results in non-invasive follicular variant papillary thyroid carcinoma cases, with a score index of 0 and quick H score of 0, as shown in Figure 5 with a magnification power of microscope 10×4.

## Discussion

In the last several decades, thyroid carcinoma has become the most rapidly developing malignancy [21]. Thyroid tumor growth incidence has long been a controversial subject among specialists. Some writers hypothesized that the observed increase in incidence was due to the increased use of precise diagnostic techniques and the identification of small subclinical tumors, which they termed overdiagnosis [22]. On the other hand, some authors hypothesized that the increased incidence is related to exposure to hormonal factors and unknown environmental carcinogens [23]. This study shows that the female gender is more predominant in PTC than males. This finding is consistent with Rashid *et al.*, who demonstrated a high female prevalence compared to males [19], and more studies agreed with this finding [24, 25]. Another study demonstrated no differences between females and males [26]. According to Jukkola *et al.*, male gender correlated with a poor prognosis, a higher rate of tumor recurrence, and a higher risk of death [27]. Furthermore, Nikiforova *et al.* found a correlation between male gender prevalence and BRAF mutation [28]. The age group in our study did not reach statistical significance (p=0.7), which agreed with other studies [29, 30]. In contrast, other studies demonstrated important benefits for the age group (mean 45) [31, 32]. In our study, there is no documented relationship with tumor size. This finding agreed with Szymonek *et al.* [33]. Also, Jian *et al.* demonstrated no association with tumor size [34], while Kim *et al.* documented tumor size as an important correlated parameter to determine tumor prognosis and recurrence [35]. Several other studies show a link between large tumor size and BRAF mutations [36, 37]. Our study found no significant relationship between BRAF V600E and tumor focality. This result is incomparable with the finding of the Sanguisi *et al.* [38], while other studies demonstrated a positive correlation between BRAF V600E and tumor focality [34].

## Conclusion

Our study demonstrates that BRAF V600E IHC is more common among malignant tumors, especially in papillary thyroid carcinoma than in other tumors. The BRAF V600E IHC mutation is strongly associated with the female rather than male gender, but it was not statistically significant. Also, BRAF V600E showed a negative relationship with age and tumor size. However, there was a strong correlation with tumor focality. BRAF V600E IHC can be used as an alternative to molecular biology to detect mutations in patients with thyroid neoplasm. This finding needs to be approved by molecular approaches, so we compared our results with previous immunohistochemistry (IHC) and molecular studies in Iraq and other countries.

## Acknowledgements

### Conflict of interest

The authors declare no conflict of interest.

### Ethical approval

This study was approved by the Medical Ethics Committee from the Faculty of Dentistry, University of Kufa (#326). 

### Consent to participate

Informed consent was obtained from all participants. 

### Authorship

MHAW contributed to data collection, analysis and writing the original paper. RHA revised the analysis and the writing and offered the final confirmation.

## References

[1] Penna GC, Vaisman F, Vaisman M, Sobrinho-Simões M, Soares P (2016). Molecular Markers Involved in Tumorigenesis of Thyroid Carcinoma: Focus on Aggressive Histotypes.. Cytogenet Genome Res..

[2] Mahdi Al-Saraj MA-M (2015). IRAQI CANCER BOARD IRAQI CANCER REGISTRY CENTER..

[3] Jung CK, Little MP, Lubin JH, Brenner AV (2014). The increase in thyroid cancer incidence during the last four decades is accompanied by a high frequency of BRAF mutations and a sharp increase in RAS mutations.. J Clin Endocrinol Metab..

[4] Hung YP, Barletta JA (2018). A user's guide to non-invasive follicular thyroid neoplasm with papillary-like nuclear features ( NIFTP ).. Natl Libr Med..

[5] Liu J, Singh B, Tallini G, Carlson DL (2006). Follicular variant of papillary thyroid carcinoma: a clinicopathologic study of a problematic entity.. Cancer..

[6] Hodak S, Tuttle RM, Maytal G, Nikiforov YE, Randolph G (2016). Changing the Cancer Diagnosis: The Case of Follicular Variant of Papillary Thyroid Cancer-Primum Non Nocere and NIFTP.. Thyroid..

[7] Rivera M, Ricarte-Filho J, Knauf J, Shaha A (2010). Molecular genotyping of papillary thyroid carcinoma follicular variant according to its histological subtypes (encapsulated *vs.* infiltrative) reveals distinct BRAF and RAS mutation patterns.. Mod Pathol..

[8] Martini M, Pantanowitz L, Thompson LDR, Larocca LM, Rossi ED (2018). A review of the cytomorphological features of NIFTP.. Diagnostic Histopathol..

[9] Thompson LD (2016). Ninety-four cases of encapsulated follicular variant of papillary thyroid carcinoma: A name change to Noninvasive Follicular Thyroid Neoplasm with Papillary-like Nuclear Features would help prevent overtreatment.. Mod Pathol..

[10] Jeon EJ, Jeong YJ, Park SH, Cho CH (2016). Ultrasonographic Characteristics of the Follicular Variant Papillary Thyroid Cancer According to the Tumor Size.. J Korean Med Sci..

[11] Zhao L, Dias-Santagata D, Sadow PM, Faquin WC (2017). Cytological, molecular, and clinical features of noninvasive follicular thyroid neoplasm with papillary-like nuclear features *versus* invasive forms of follicular variant of papillary thyroid carcinoma.. Cancer Cytopathol..

[12] Zhi J, Zhao J, Gao M, Pan Y (2018). Impact of major different variants of papillary thyroid microcarcinoma on the clinicopathological characteristics: the study of 1041 cases.. Int J Clin Oncol..

[13] Siddiqui S, White MG, Antic T, Grogan RH (2016). Clinical and Pathologic Predictors of Lymph Node Metastasis and Recurrence in Papillary Thyroid Microcarcinoma.. Thyroid..

[14] Paulson VA, Shivdasani P, Angell TE, Cibas ES (2017). Noninvasive Follicular Thyroid Neoplasm with Papillary-Like Nuclear Features Accounts for More Than Half of "Carcinomas" Harboring RAS Mutations.. Thyroid..

[15] Bongiovanni M, Faquin WC, Giovanella L, Durante C (2019). Impact of non-invasive follicular thyroid neoplasms with papillary-like nuclear features (NIFTP) on risk of malignancy in patients undergoing lobectomy/thyroidectomy for suspected malignancy or malignant fine-needle aspiration cytology findings: a systematic review and meta-analysis.. Eur J Endocrinol..

[16] Pongsapich W, Chongkolwatana C, Poungvarin N, Amornpichetkul K (2019). BRAF mutation in cytologically indeterminate thyroid nodules: after reclassification of a variant thyroid carcinoma.. Onco Targets Ther..

[17] Capper D, Preusser M, Habel A, Sahm F (2011). Assessment of BRAF V600E mutation status by immunohistochemistry with a mutation-specific monoclonal antibody.. Acta Neuropathol..

[18] Cabanillas ME, Dadu R, Iyer P, Wanland KB (2020). Acquired Secondary RAS Mutation in BRAFV600E-Mutated Thyroid Cancer Patients Treated with BRAF Inhibitors.. Thyroid..

[19] Rashid FA, Tabassum S, Khan MS, Ansari HR (2021). VE1 immunohistochemistry is an adjunct tool for detection of BRAFV600E mutation: Validation in thyroid cancer patients.. J Clin Lab Anal..

[20] Zaballos MA, Santisteban P (2017). Key signaling pathways in thyroid cancer.. J Endocrinol..

[21] Siegel RL, Miller KD, Jemal A (2016). Cancer statistics, 2016.. CA Cancer J Clin..

[22] O'Grady TJ, Gates MA, Boscoe FP (2015). Thyroid cancer incidence attributable to overdiagnosis in the United States 1981-2011. Int J Cancer..

[23] Morris LG, Myssiorek D (2010). Improved detection does not fully explain the rising incidence of well-differentiated thyroid cancer: a population-based analysis.. Am J Surg..

[24] Salih AM, Naqshabandi MM, Hassan NA (2017). Braf Gene Mutation and Cd56 Immunoexpression in Papillary Thyroid Carcinoma in Duhok-Iraq.. J Sulaimani Med Coll..

[25] Min HS, Lee C, Jung KC (2013). Correlation of immunohistochemical markers and BRAF mutation status with histological variants of papillary thyroid carcinoma in the Korean population.. J Korean Med Sci..

[26] Kareem MA, Jasim Mohamad B (2017). Detection of Pan Braf in Thyroid Tumors in Iraqi Patients.. Iraqi J Sci..

[27] Jukkola A, Bloigu R, Ebeling T, Salmela P, Blanco G (2004). Prognostic factors in differentiated thyroid carcinomas and their implications for current staging classifications.. Endocr Relat Cancer..

[28] Nikiforova MN, Kimura ET, Gandhi M, Biddinger PW (2003). BRAF mutations in thyroid tumors are restricted to papillary carcinomas and anaplastic or poorly differentiated carcinomas arising from papillary carcinomas.. J Clin Endocrinol Metab..

[29] Kristiani E, Hardjolukito ES, Harahap AS, Makes B (2021). BRAF V600E Immunoexpression in Papillary Thyroid Carcinoma and Its Association with Prognostic Factors and Histopathologic Variant.. Medicinus..

[30] Abd Elmageed ZY, Sholl AB, Tsumagari K, Al-Qurayshi Z (2017). Immunohistochemistry as an accurate tool for evaluating BRAF-V600E mutation in 130 samples of papillary thyroid cancer.. Surgery..

[31] Zagzag J, Pollack A, Dultz L, Dhar S (2013). Clinical utility of immunohistochemistry for the detection of the BRAF v600e mutation in papillary thyroid carcinoma.. Surgery..

[32] McKelvie PA, Chan F, Yu Y, Waring P (2013). The prognostic significance of the BRAF V600E mutation in papillary thyroid carcinoma detected by mutation-specific immunohistochemistry.. Pathology..

[33] Szymonek M, Kowalik A, Kopczyński J, Gąsior-Perczak D (2017). Immunohistochemistry cannot replace DNA analysis for evaluation of BRAF V600E mutations in papillary thyroid carcinoma.. Oncotarget..

[34] Sun J, Zhang J, Lu J, Gao J (2015). Immunohistochemistry is highly sensitive and specific for detecting the BRAF V600E mutation in papillary thyroid carcinoma.. Int J Clin Exp Pathol..

[35] Kim SJ, Lee KE, Myong JP, Park JH (2012). BRAF V600E mutation is associated with tumor aggressiveness in papillary thyroid cancer.. World J Surg..

[36] Kwak JY, Kim EK, Chung WY, Moon HJ (2009). Association of BRAFV600E mutation with poor clinical prognostic factors and US features in Korean patients with papillary thyroid microcarcinoma.. Radiology..

[37] Kim KH, Suh KS, Kang DW, Kang DY (2005). Mutations of the BRAF gene in papillary thyroid carcinoma and in Hashimoto's thyroiditis.. Pathol Int..

[38] Sancisi V, Nicoli D, Ragazzi M, Piana S, Ciarrocchi A (2012). BRAFV600E mutation does not mean distant metastasis in thyroid papillary carcinomas.. J Clin Endocrinol Metab..

